# Emergency food distribution efforts in New Orleans, LA after Hurricane Ida

**DOI:** 10.3389/fpubh.2022.968552

**Published:** 2022-09-07

**Authors:** Chelsea R. Singleton, M. Pia Chaparro, Keelia O'Malley, Melissa Fuster, Donald Rose

**Affiliations:** Department of Social, Behavioral, and Population Sciences, Tulane School of Public Health and Tropical Medicine, New Orleans, LA, United States

**Keywords:** hurricane, natural disaster, poverty, equity, food aid, New Orleans

## Abstract

**Background:**

The provision of food aid after a natural disaster is necessary to prevent hunger, particularly in low-resourced and low-income communities. Little is known about the operational challenges associated with ensuring equitable distribution of emergency food resources to communities in need following a disaster. To address this gap, this study assessed emergency food distribution efforts in New Orleans, LA during the 2 weeks following Hurricane Ida's landfall on August 29, 2021.

**Methods:**

Information on free food distribution events was gathered from online sources. A list of distribution sites was generated that included data on operational logistics (e.g., address, days of operation, hours of operation, etc.), food offerings (e.g., prepared meals, groceries, etc.), and socio-demographic characteristics of the surrounding community. Geospatial mapping and bivariate analyses were used to analyze the site data.

**Results:**

Seventy-four distribution sites operated in the 2 weeks after Hurricane Ida. Approximately 47.3% were located in census tracts with >80% Black residents, and 39.2% were in tracts with >30% poverty. A large proportion of sites offered prepared meals (86.2%) and only operated 1 day (36.5%). Tracts with >80% Black residents had more sites that operated only 1 day (*p* = 0.04). Tracts with >30% poverty had more sites that started distributing food resources 7–15 days after the hurricane (*p* = 0.02).

**Conclusions:**

Most low-income and low-resourced communities in New Orleans had access to emergency food resources; however, several limitations in operations were identified that may have influenced access. Future initiatives to prevent hunger after a natural disaster in New Orleans, and elsewhere, should improve operational logistics for food aid.

## Introduction

Hurricane Ida made landfall in Louisiana (LA) as a category 4 storm on August 29, 2021, 16 years, to the date, after Hurricane Katrina (1). While the effects were not as severe or long lasting as Katrina's, Ida caused significant damage to the physical infrastructure and environment in New Orleans, LA (2). The damage to the city's power grid reported by the region's energy supplier surpassed what they found after Katrina (2). More than a million customers lost power (3). As a result, most residents and food retail businesses lost their entire food supply as power outages persisted in the 2 weeks following Ida. High summer temperatures coupled with limited food resources increased concern among community leaders and city officials about hunger and dehydration (4).

In response, several non-profit organizations, food retail businesses, and city officials rallied to establish emergency food distribution sites throughout New Orleans (4–6). Emergency food assistance is a key aspect of a society's ability to respond to natural disasters (7, 8). Along with water and shelter, food is a basic need, and its absence jeopardizes a city's ability to advance recovery efforts (7). Therefore, recovery plans for natural disasters should ensure food assistance is provided to residents in a safe and equitable manner (8).

Prior research has shown that socially and economically disadvantaged populations are more likely to experience the detrimental effects of natural disasters (7–11). Low-income individuals and families may lack the necessary financial resources, social connections, and transportation to evacuate prior to a hurricane, or immediately afterwards (8). Thus, this population is more likely to remain in their homes and wait out the storm. In doing so, they increase their risk of serious physical injury and other adverse health outcomes associated with experiencing the aftermath of a major hurricane, including food insecurity (8, 9). To ensure food assistance resources are equitably distributed in cities after a natural disaster, the field needs up-to-date quantitative information on the successes and challenges of existing emergency food distribution efforts in disaster prone regions.

The overarching aim of this study was to evaluate emergency food distribution efforts in New Orleans, LA in the 2 weeks following Hurricane Ida's landfall. Specifically, this study aimed to (1) identify the types of food assistance resources made available to New Orleans residents post-Ida; (2) determine if access to resources varied by community-level socio-demographic indicators; and (3) present evidence-based recommendations that can inform future hurricane preparedness plans. This research is necessary and timely. Hurricane Ida is just one of many natural disasters to affect the Gulf Coast region of the U.S. in recent decades. Since climate change experts predict the frequency and severity of these disasters will increase in the near future, it is imperative that cities in regions prone to disasters strengthen their preparedness plans and emergency food efforts (12).

## Methods

### Data sources

**Figure 1:**
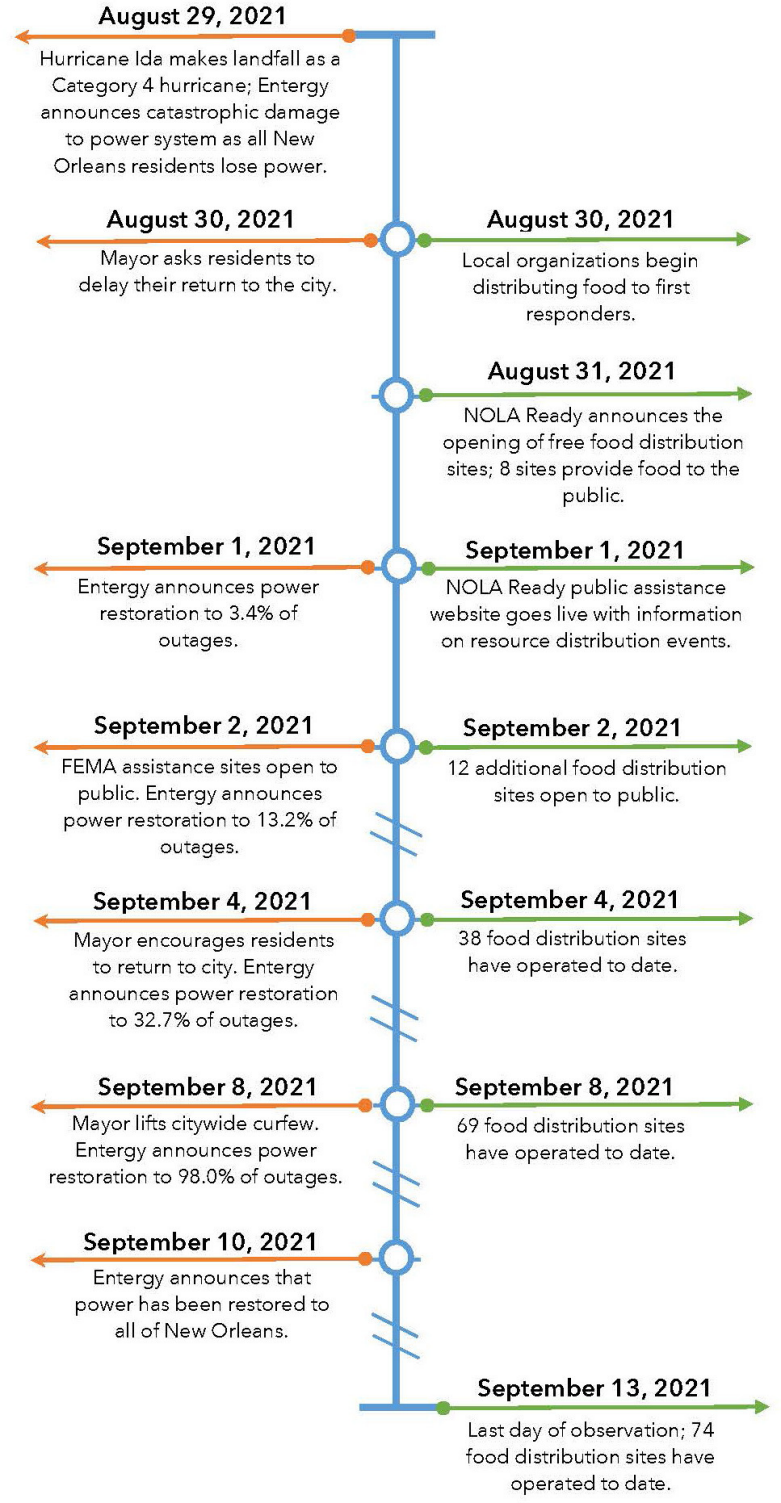
Timeline of Hurricane Ida Recovery and Food Aid Response in New Orleans, LA between 8/29/21 and 9/13/21. Timeline of events developed by authors. Dates and events presented reflect publicly available information published by Entergy and *NOLA Ready* (2, 3, 5, 6).

The institutional review board at Tulane University deemed this exempt research. To address the study aims, members of the research team began tracking and gathering publicly available data on food distribution events held in New Orleans, LA after Hurricane Ida. They gathered data on all events that occurred during the 2 weeks following the storm (August 30-September 13). This timeline was selected because distribution events greatly reduced in frequency after September 13, since power had been restored to all residences and most commercial businesses had re-opened. Figure 1 presents a complete timeline of the food aid response and recovery milestones in New Orleans following the hurricane.

A comprehensive list of food distribution events was developed after analyzing data from three sources: (1) the *NOLA Ready* public assistance calendar, (2) press releases published by nola.com, a local news outlet, in the 48 h following the hurricane, and (3) community leaders (5, 6). On September 1, *NOLA Ready*, the public-facing website for New Orleans' Office of Homeland Security and Emergency Preparedness, published an interactive calendar that provided the dates, times, and locations where residents could receive free resources (e.g., food, water, ice, tarps, cooling, electronic device charging, etc.) (6). The calendar only included information on resource distribution events in the city. Events in neighboring cities or parishes were not included. The *NOLA Ready* assistance reporting system was passive; organizers and businesses had to contact *NOLA Ready* to include their food distribution events on the calendar. Thus, information provided by the other two data sources was included to ensure the development of an accurate list of events.

In the 48 h prior to the calendar going live (August 30–31), *NOLA Ready* published press releases on food distribution events *via* nola.com (5). All relevant nola.com press releases posted online on those days were examined to extract relevant information on events. Research team members contacted leaders of several community organizations mentioned by local media outlets as hosts of food distribution events. If the *NOLA Ready* public assistance calendar did not include their event, leaders were asked to supply the address, hours of operation, and item distribution information for every day the organization distributed free food to the public.

### Data extraction

Using the data gathered and Google Maps, two trained research assistants drafted a comprehensive list of food distribution events. An event was eligible for inclusion if it distributed free food to the public and was held between August 30 and September 13. Events that solely distributed resources such as water or ice were excluded. For each event, the following variables were extracted: date of event, address, longitude of address, latitude of address, hours of operation, location of event (e.g., community center, church, school, etc.), meal ready-to-eat (MRE) distribution status (yes or no), prepared meal distribution status (yes or no), grocery distribution status (yes or no), water distribution status (yes or no), and ice distribution status (yes or no). Hours of operation were categorized to reflect the following periods: morning (8 am-noon), afternoon (noon-4pm), evening (after 4pm), multiple (two of the aforementioned periods), and all day. A categorical variable representing first date of operation was created to identify events that occurred within days 1–3 (August 30–September 1), days 4–6 (September 2–4), and days 7–15 (September 5–13) after the hurricane.

An MRE was defined as a small, packaged meal ration that may or may not be dehydrated or freeze-dried. These meals, which are often used in by the military, have a long shelf life. Prepared meals included any hot or cold meal that residents could eat on the premises or take away. For most events listed on the *NOLA Ready* calendar, the organizers did not provide details about the meals served. Therefore, no criteria were set for meal size, type, or quality. Free groceries were defined as free perishable (e.g., raw meat, milk, vegetables, or bread) or non-perishable (e.g., canned goods) food items that residents must cook and consume at home. Only events that gave out water and/or ice in bulk (e.g., bags, jugs, or cases) received a “yes” for water and ice distribution. Single servings of water or ice provided with free meals did not count.

### Geospatial mapping and statistical analysis

Data on food distribution events were grouped by location address to identify unique food distribution sites throughout the city. ArcGIS software was used to map the addresses of distribution sites over census tract-level shape-files representing % non-Hispanic (NH) Black residents and % poverty (13). Stata software was used to examine census-tract level associations between various distribution site characteristics and census tract characteristics (i.e., % NH Black, % poverty, and low-income/low-access status) (14). Data on % NH Black and % poverty were extracted from the U.S. Census Bureau and represent 5-year American Community Survey (ACS) estimates for 2019 (15). Data on low-income/low-access status for all census tracts were obtained from the 2019 U.S. Department of Agriculture (USDA) Food Access Atlas (16). The USDA labels a census tract as “low-income/low-access” if ≥ 20% of residents live in poverty and ≥ 33% of residents live more than one-half mile (urban areas) from the nearest supermarket, supercenter, or large grocery store (17). Frequencies were tabulated for variables representing site operations and resource offerings among all distribution sites and stratified by days of operation (1 day, 2–7 days, and 8+ days), % NH Black residents (<60, 60–80, and >80), and % poverty (<20, 20–30, >30). Fisher's Exact test evaluated bivariate associations between variables of interest and the categorical variables for days of operation, % NH Black, and % poverty. *P* < 0.05 were considered statistically significant.

## Results

**Figure 2:**
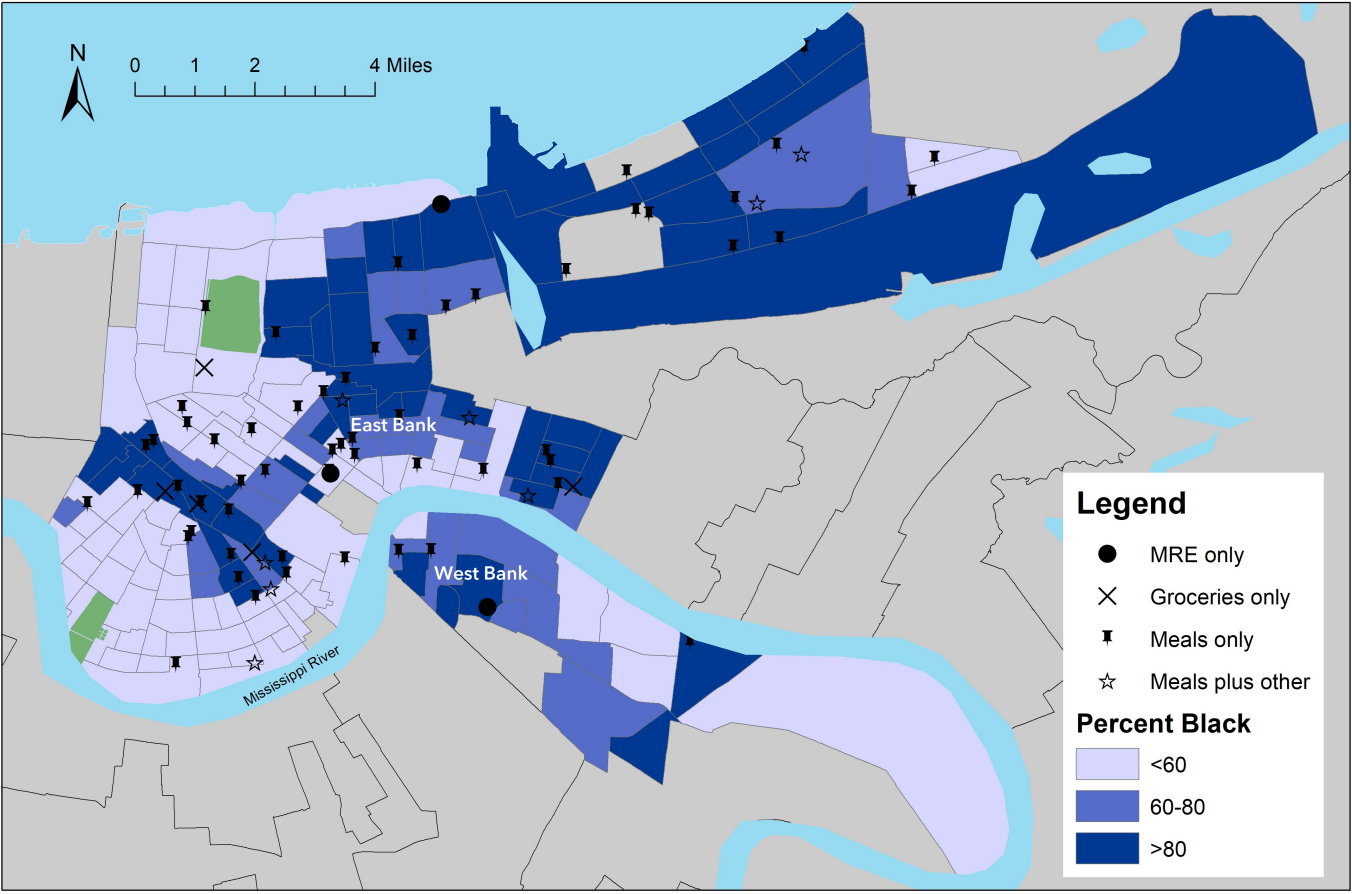
Location of Emergency Food Distribution Sites in New Orleans Relative to Census Tract-Level Estimates for % NH Black Residents. Data on sites locations provided by *NOLA Ready*, the city's emergency preparedness office. Census tract-level estimates of % NH Black residents were obtained from the U.S. Census Bureau (6, 15).

**Figure 3:**
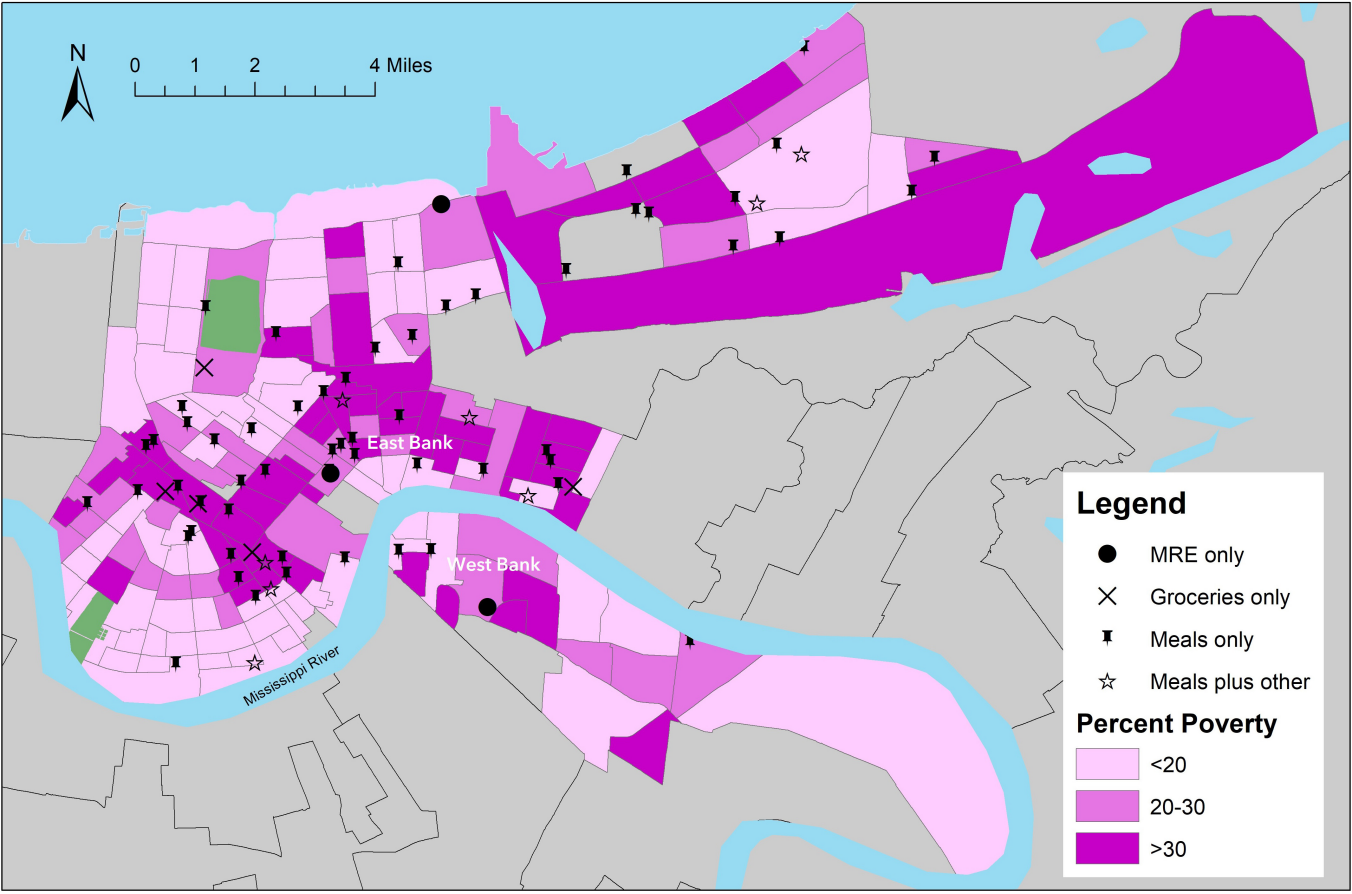
Location of Emergency Food Distribution Sites in New Orleans Relative to Census Tract-Level Estimates for % Poverty. _Data on sites locations provided by *NOLA Ready*, the city's emergency preparedness office. Census tract-level estimates of % poverty were obtained from the U.S. Census Bureau (6, 15).

Seventy-four food distribution sites were identified during the two-week observation period. Panel maps presented in Figures 2, 3 display geographic locations of sites relative to the census-tract level estimates for % NH Black and % poverty, respectively. Sites offerings are labeled to identify sites that distributed prepared meals only, MREs only, groceries only, or a combination of prepared meals and at least one other food resource (MREs or groceries). The maps revealed that most tracts in New Orleans with high percentages of low-income and NH Black residents had sufficient access to distribution sites, particularly those offering prepared meals. A gap in access was visually identified on the West Bank neighborhoods of the city, which had several high-poverty census tracts but few distributions sites.

Information on socio-demographics, site operations, and site resources are recorded in Table 1 stratified by total days of operation. There were 27 (36.5%), 23 (31.1%), and 24 (32.4%) sites that operated one day, 2–7 days, and 8+ days, respectively. A large proportion of sites (47.3%) were located in census tracts where >80% of the residents were NH Black. Thirty-five sites (39.2%) were in tracts with a % poverty >30%, which is higher than the city's median for persons in poverty for 2019 (i.e., 23.7%) (15). Most sites (54.1%) were in a USDA-designated low-income/low-access census tract.

**Table 1:** Food distribution site demographics, operations, and resources by total days of operation, N (%).^a^

**Variable:**	**All sites**	**1 Day**	**2–7 Days**	**8+ Days**	***P* Value^b^**
	***N* = 74**	**27 (36.5)**	**23 (31.1)**	**24 (32.4)**	
**Socio-demographics** ^**c**^					
% NH Black:					0.04
<60	22 (29.7)	3 (11.1)	10 (43.5)	9 (37.5)	
60–80	17 (23.0)	10 (37.0)	2 (8.7)	5 (20.8)	
>80	35 (47.3)	14 (51.9)	11 (47.8)	10 (41.7)	
% Poverty:					0.55
<20	25 (33.8)	8 (29.6)	10 (43.5)	7 (29.2)	
20–30	20 (27.0)	6 (22.2)	5 (21.7)	9 (37.5)	
>30	29 (39.2)	13 (48.2)	8 (34.8)	8 (33.3)	
Low-income/low-access	40 (54.1)	19 (70.4)	11 (47.8)	10 (41.7)	0.10
					
**Site operations** ^**d**^					
Morning	1 (0.1)	1 (3.7)	0 (0.0)	0 (0.0)	-
Afternoon	38 (51.4)	11 (40.7)	12 (52.2)	15 (62.5)	0.32
Evening	23 (31.1)	8 (29.6)	5 (21.7)	10 (41.7)	0.34
Multiple^e^	29 (39.2)	5 (18.5)	12 (52.2)	12 (50.0)	0.02
All day	8 (10.8)	1 (3.7)	1 (4.4)	6 (25.0)	0.04
First date of operation:					<0.0001
Day 1–3	28 (37.8)	5 (18.5)	4 (17.4)	19 (79.2)	
Day 4–6	26 (35.1)	9 (33.3)	12 (52.2)	5 (28.8)	
Day 7–15	20 (27.0)	13 (48.2)	7 (30.4)	0 (0.0)	
**Site type:**					
Community center	22 (29.7)	3 (11.1)	8 (34.8)	11 (45.8)	0.13
Church	20 (27.0)	11 (40.7)	5 (21.7)	4 (16.7)	
School/park/public street	18 (24.3)	8 (29.6)	6 (26.1)	4 (16.7)	
Food retailer/other^f^	14 (18.9)	5 (18.5)	4 (17.4)	5 (20.8)	
					
**Site resources** ^**d**^					
Prepared meals	66 (89.2)	23 (85.2)	22 (91.7)	21 (87.5)	0.52
MREs	7 (9.5)	1 (3.7)	0 (0.0)	6 (25.0)	0.007
Groceries	9 (12.2)	5 (18.5)	3 (13.0)	1 (4.2)	0.30
Water	28 (37.8)	5 (18.5)	7 (30.4)	16 (66.7)	0.002
Ice	10 (13.5)	2 (7.4)	1 (4.4)	7 (29.2)	0.04

NH, Non-Hispanic; MRE, meals ready-to-eat.

^a^Data presented in this table represents aggregate information on emergency food distribution sites that operated in the 2 weeks after Hurricane Ida (August 30–September 13, 2021).

^b^P values calculated with Fisher's Exact test. Values < 0.05 were considered statistically significant.

^c^Socio-demographic data represent estimates of the population in the census tracts where the food distribution sites were located. These data were gathered from the U.S. Census Bureau (% NH Black and % poverty) or the U.S. Department of Agriculture's Food Access Atlas (low-income/low-access census tract).

^d^Site operation and resource data were extracted from the *NOLA Ready* public assistance calendar. Variables representing site operations and resources are not mutually exclusive.

^e^Multiple indicates that a site was open during two periods or the day: morning/afternoon, morning/evening, or afternoon/evening.

^f^Other distribution site includes event/entertainment centers and low-income housing complexes.

Eight sites (10.8%) distributed food all day at least once during the observation period. The most common time period of operation reported was afternoon, which represented 38 sites (51.4%). It is important to note that several sites changed hours of operation over the two-week period. Thus, categories for hours of operation are not mutually exclusive. Twenty-eight sites (37.8%) reported their first day of operation within 3 days after the storm, and distribution sites were primarily community centers (29.7%) and churches (27.0%). The vast majority of sites offered prepared meals at least once during the observation period (89.2%). MREs and groceries were only offered at 7 (9.5%) and 9 (12.2%) sites, respectively. Water was offered at 28 (37.8%) distribution sites.

Several variables were associated with total days of operation including % NH Black residents (p = 0.04). About half of the sites that operated only one day were in census tracts with > 80% NH Black residents whereas over half of the sites that operated 8+ days were in tracts with ≤ 80% NH Black residents. About 48% of sites that operated one day did so between 7 and 15 days after the hurricane (*p* < 0.0001). Most of the sites that operated multiple periods during the day (*p* = 0.02) or all day (*p* = 0.04) reported being open 8+ days. In addition, most of the sited offering MREs (*p* = 0.007), water (*p* = 0.002), and ice (*p* = 0.04) operated 8+ days.

Supplementary Tables 1, 2 present site information by % NH Black and % poverty, respectively. A significant association was detected between % poverty and first date of operation (*p* = 0.02). Compared to low poverty census tracts (<20%), a greater percentage of sites in census tracts with >30% poverty had a first date of operation 7–15 days after the hurricane.

## Discussion

This study aimed to describe the food aid response in New Orleans, LA in the 2 weeks following Hurricane Ida's landfall in order to identify opportunities to improve emergency preparedness plans. While this research solely focuses on the post-Ida response in New Orleans, LA, the findings can be applicable to planning efforts in other areas of the U.S. and countries with similar propensity to hurricanes. Furthermore, local governments and communities can use the recommendations identified to strengthen support for emergency food assistance after a natural disaster.

Overall, results indicated that New Orleans residents had several locations where they could access emergency food resources. The vast majority of distribution sites were in census tracts with high percentages of low-income and NH Black residents, which is particularly important given existing knowledge of post-Katrina inequities in food security and food access (18–22). Food insecurity continues to be a major public health issue in New Orleans, the State of Louisiana, and the Gulf Coast region (18–20). According to Feeding America, the prevalence of food insecurity in Louisiana is about 16%, which is higher than the national average (≈ 11%) (18). A longitudinal study by Clay et al. (18) revealed that low-income and minority status were significantly associated with continued risk of food insecurity among Gulf Coast residents after Katrina (19). Longitudinal assessments of food access disparities in post-Katrina New Orleans revealed similar results (20, 21). Studies by Rose et al. (20) and Mundorf et al. (21) reported that food access, specifically access to supermarkets, worsened in the city's Black communities after Katrina (20, 21). To ensure equitable response and recovery after a natural disaster, it is important to provide sufficient access to resources in communities that historically have endured more adverse effects and slower recovery.

Despite the relatively equitable availability of site locations, a close examination of site operations revealed some areas of concerns. Total days of operation was negatively associated with percent of NH Black residents. Forty percent of distributions sites in census tracts with >80% NH Black residents were only open for 1 day. This compares to only 13.6% of sites in tracts with <60% NH Black residents. Although not a statistically significant association, it is important to note that half of the 40 distribution sites in low-income/low-access census tracts only operated 1 day. Moreover, high-poverty census tracts had a greater percentage of sites that opened a week after the storm, denoting a potential lack of food resources in the crucial days immediately following the hurricane's landfall. Overall, these findings demonstrate the importance of considering factors beyond geographic access of site locations, including operations, resource offerings, etc., when evaluating emergency aid distribution after a natural disaster. Although prior studies have evaluated the efficacy of emergency food distribution efforts after natural disasters in the U.S. and abroad, they have not applied an equity lens to these efforts (22–26). Future studies aiming to assess the provision of food aid after a natural disaster should thoroughly evaluate site operations, logistics, and offerings to identify areas of improvement and reduce inequities.

This research has strengths and limitations. The approach to data gathering was a strength. To ensure the creation of a robust list of food distribution sites, data on distribution events were collected from *NOLA Ready*, official news sources, and leaders of local community organizations with reputations for providing food aid. *NOLA Ready*, the city's emergency management office, compiled and advertised information on most food distribution events included in the study sample (5, 6). Limitations include the sample size and passive reporting system on resource availability established by *NOLA Ready*. Because the analytical sample comprised only 74 sites, it limited statistical power and the ability to detect statistically significant associations. Individuals and organizations that distributed food had to contact *NOLA Ready* to post their distribution event on the assistance calendar. There were organizations that began distributing food to first responders, assisted living centers, and low-income housing complexes on the day after the Hurricane Ida. Unfortunately, information about these events are not available to the public. Therefore, it is likely that this study's sample of food distribution events did not include all events held in the city after Ida. Another limitation was the data source, which lacked detailed information on food offerings and event sponsors. No data was available on the total number of meals distributed per day at each site. If available, these data would have provided city officials and local organizations greater understanding of community need and the effectiveness of distribution site placement. Data on the quantity and types of foods offered at each event were also not available, preventing an assessment of the nutritional quality of foods distributed. Furthermore, no definite information on the individuals and organizations that sponsored the events were available. These data would have provided much needed insight on the source(s) of funding for each event (e.g., the city, community organization, private business, etc.).

## Recommendations for preparedness reform

This study found that many low-income and low-resourced communities in New Orleans had access to emergency food resources after Hurricane Ida. However, operational concerns were identified that have potential to influence access. These findings have strong implications for emergency preparedness reform and underscore the need to strengthen the food aid response in New Orleans, LA following a major natural disaster. Evidence-based recommendations for reform identified by members of this study's research team are grouped and described below as follows: (1) data collection, (2) resource offerings, (3) capacity building, and (4) nutrition equity.

### Data collection

Appropriate methods for data collection are needed to ensure accurate reporting on emergency resources. In New Orleans, the emergency management office utilized a passive data collection system to gather information on food distribution events after Ida. Event details are submitted by sponsors and advertised to residents *via* the city's website, social media, and local news outlets (5, 6). In addition, text messages about resources are sent to mobile devices; however, residents must sign up to receive these messages. This approach to information collection places the burden of event advertisement on sponsors, which can result in events not being widely advertised. Communications after a natural disaster are critical to ensuring local residents are connected to emergency resources in a timely manner (23). A study by Sudo et al. from Japan reported that local municipalities previously effected by a natural disaster were more likely to have established systems to collect up-to-date information from organizations and shelters (23). Emergency management officials should allocate resources (i.e., funds, personnel) to establish systems capable of collecting standardized information on distribution events directly from sponsors. Since many organizations begin planning their efforts in the days prior to the storm, the system should begin compiling data prior to disaster, if possible.

### Resource offerings

The data sources utilized for this research did not provide in-depth information on the food resources offered at each distribution event. Thus, no data were available on the size, quantity, and quality of meals or the total amount of meals distributed per day at each site. Future initiatives to improve distribution efforts should consider reporting real-time information on the types of meals served at events and the amount of individuals and families served per day. This will (1) allow local residents to determine if these meals meet their nutritional needs (2) facilitate in-depth evaluation of the nutritional quality of food resources, and (3) permit the tracking of each food distribution site's day-to-day activities in regards to supply and demand. Prior studies have investigated the nutritional quality of emergency meals distributed after a natural disaster (22, 24–26). For example, Colón-Ramos et al. (22) reported that the meals distributed at a federal distribution site in Puerto Rico after Hurricane Maria did not align with the Dietary Guidelines for Americans (22). A large number of community organizations distributed food in New Orleans after Ida, so it is likely that the nutritional quality of meals varied greatly by site and day. Given the typical disruptions that occur after a natural disaster (e.g., transportation, power, social services), it is important to provide public information on the meals offered at distribution sites so residents can make informed decisions.

### Capacity building

There were at least 74 locations in New Orleans where residents could access food resources after Ida. Local community organizations sponsored the vast majority of the distribution events, which demonstrates the grassroots nature of the post-disaster food aid response in New Orleans. City officials can build capacity for emergency food distribution by allocating resources that will centralize and support the efforts of these groups. Ainehvand et al. used qualitative methods to conduct a thorough evaluation of challenges associated with providing food resources after natural disasters in Iran (24). They identified several challenges with coordination and communication among government and non-government entities, which ultimately resulted in parallel and repetitive practices (24). Centralizing efforts and increasing support for local organizations and businesses may result in better coordination of event locations, logistics, and offerings across the city. Of particular importance, given the widespread power outages, is equipping local organizations that can provide prepared meals, such as schools or restaurants, with alternative energy sources (e.g., solar panels, generators).

### Nutrition equity

While findings from this study revealed relatively sufficient access to food distribution sites in New Orleans after Ida, analyses of site logistics revealed key inequities in the food aid response. Like other public crises, natural disasters can reveal, and worsen, inequities in health and resource availability (7–9, 27, 28). Ida occurred during a global pandemic that has disproportionately increased unemployment and food insecurity rates among low-income individuals and racial/ethnic minorities at the national and local levels (29, 30). Policies and initiatives implemented to reduce food insecurity since the onset of COVID-19 pandemic have stressed the importance of nutrition equity (31, 32). Future efforts to strengthen the food aid response in New Orleans and areas prone to disasters should emphasize nutrition equity by targeting low-income and low-resourced communities. This requires applying an equity lenses to data collection, communications, event marketing, logistics, and resource offerings.

## Data availability statement

The original contributions presented in the study are included in the article/Supplementary material, further inquiries can be directed to the corresponding author.

## Author contributions

CS and KO performed the data gathering and statistical analysis. MC performed the mapping. CS developed the initial draft of the manuscript. All authors conceptualized this research project, edited the paper, and approved the final draft for publication.

## Conflict of interest

The authors declare that the research was conducted in the absence of any commercial or financial relationships that could be construed as a potential conflict of interest.

## Publisher's note

All claims expressed in this article are solely those of the authors and do not necessarily represent those of their affiliated organizations, or those of the publisher, the editors and the reviewers. Any product that may be evaluated in this article, or claim that may be made by its manufacturer, is not guaranteed or endorsed by the publisher.
