# Supplementary material for: Emergency food distribution efforts in New Orleans, LA after Hurricane Ida
Source: Front Public Health. 2022 Sep 7;10:968552. doi: 10.3389/fpubh.2022.968552 (PMC9489999; doi:10.3389/fpubh.2022.968552)
Supplement: Supplementary file 1 [file Data_Sheet_1.docx]

**Supplemental Table 1** – Food Distribution Site Demographics, Operations, and Resources by % NH Black in the Surrounding Census Tract.^a^

| **Variable:** | **All Sites** | **<60%** | **60-80%** | **>80%** | ***P* Value^b^** |
| --- | --- | --- | --- | --- | --- |
|  | **N = 74** | **22 (29.7)** | **17 (23.0)** | **35 (47.3)** |  |
| *Socio-Demographics^c^* |  |  |  |  |  |
| % Poverty: |  |  |  |  | <0.0001 |
| <20 | 25 (33.8) | 10 (45.5) | 11 (64.7) | 4 (11.4) |  |
| 20-30 | 20 (27.0) | 11 (50.0) | 1 (5.9) | 8 (22.9) |  |
| >30 | 29 (39.2) | 1 (4.6) | 5 (29.4) | 23 (65.7) |  |
| Low-Income/Low-Access | 40 (54.1) | 6 (27.3) | 6 (35.3) | 28 (80.0) | <0.0001 |
|  |  |  |  |  |  |
| *Site Operations^d^* |  |  |  |  |  |
| Morning | 1 (0.1) | 0 (0.0) | 0 (0.0) | 1 (2.9) | - |
| Afternoon | 38 (51.4) | 13 (59.1) | 7 (41.2) | 18 (51.4) | 0.54 |
| Evening | 23 (31.1) | 9 (40.9) | 3 (17.7) | 11 (31.4) | 0.32 |
| Multiple^e^ | 29 (39.2) | 9 (40.9) | 4 (23.5) | 16 (45.7) | 0.31 |
| All Day | 8 (10.8) | 2 (9.1) | 3 (17.7) | 3 (8.6) | 0.62 |
| Total Days of Operation: |  |  |  |  |  |
| 1 Day | 27 (36.5) | 3 (13.6) | 10 (58.8) | 14 (40.0) | 0.04 |
| 2-7 Days | 23 (31.1) | 10 (45.5) | 2 (11.8) | 11 (31.4) |  |
| 8+ Days | 24 (32.4) | 9 (40.9) | 5 (29.4) | 10 (28.6) |  |
| First Date of Operation: |  |  |  |  | 0.48 |
| Day 1 - 3 | 28 (37.8) | 11 (50.0) | 6 (35.3) | 11 (31.4) |  |
| Day 4 - 6 | 26 (35.1) | 8 (36.4) | 6 (35.3) | 12 (34.3) |  |
| Day 7 - 15 | 20 (27.0) | 3 (13.6) | 5 (29.4) | 12 (34.3) |  |
| Site Type: |  |  |  |  |  |
| Community Center | 22 (29.7) | 8 (36.4) | 5 (29.4) | 9 (25.7) | 0.17 |
| Church | 20 (27.0) | 3 (13.6) | 2 (11.8) | 15 (42.9) |  |
| School/Park/Public Street | 18 (24.3) | 6 (27.3) | 6 (35.3) | 6 (17.1) |  |
| Food Retailer/Other^f^ | 14 (18.9) | 5 (22.7) | 4 (23.5) | 5 (14.3) |  |
|  |  |  |  |  |  |
| *Site Resources^d^* |  |  |  |  |  |
| Prepared Meals | 66 (89.2) | 20 (90.9) | 16 (94.1) | 30 (85.7) | 0.71 |
| MREs | 7 (9.5) | 2 (9.1) | 3 (17.7) | 2 (5.7) | 0.40 |
| Groceries | 9 (12.2) | 1 (4.6) | 1 (5.9) | 7 (20.0) | 0.22 |
| Water | 28 (37.8) | 7 (31.8) | 5 (29.4) | 16 (45.7) | 0.47 |
| Ice | 10 (13.5) | 3 (13.6) | 3 (17.7) | 4 (11.4) | 0.91 |

Abbreviations: NH, Non-Hispanic; MRE, meals ready-to-eat.

1. Data presented in this table represents aggregate information on emergency food distribution sites that operated in the two weeks after Hurricane Ida (August 30 – September 13, 2021).
2. *P* values calculated with Fisher’s Exact test. Values < 0.05 were considered statistically significant.
3. Socio-demographic data represent estimates of the population in the census tracts where the food distribution sites were located. These data were gathered from the U.S. Census Bureau (% NH Black and % poverty) or the U.S. Department of Agriculture’s Food Access Atlas (low-income/low-access census tract).
4. Site operation and resource data were extracted from the NOLA Ready public assistance calendar. Variables representing site operations and resources are not mutually exclusive.
5. Multiple indicates that a site was open during two periods or the day: morning/afternoon, morning/evening, or afternoon/evening.
6. Other distribution site includes event/entertainment centers and low-income housing complexes.

**Supplemental Table 2** – Food Distribution Site Demographics, Operations, and Resources by % Poverty in the Surrounding Census Tract.^a^

| **Variable:** | **All Sites** | **<20%** | **20-30%** | **>30%** | ***P* Value^b^** |
| --- | --- | --- | --- | --- | --- |
|  | **N = 74** | **25 (33.8)** | **20 (27.0)** | **29 (39.2)** |  |
| *Socio-Demographics^c^* |  |  |  |  |  |
| % NH Black: |  |  |  |  | <0.0001 |
| <60 | 22 (29.7) | 10 (40.0) | 11 (55.0) | 1 (3.5) |  |
| 60-80 | 17 (23.0) | 11 (44.0) | 1 (5.0) | 5 (17.2) |  |
| >80 | 35 (47.3) | 4 (16.0) | 8 (40.0) | 23 (79.3) |  |
| Low-Income/Low-Access | 40 (54.1) | 6 (24.0) | 15 (75.0) | 19 (65.5) | 0.001 |
|  |  |  |  |  |  |
| *Site Operations^d^* |  |  |  |  |  |
| Morning | 1 (0.1) | 0 (0.0) | 0 (0.0) | 1 (3.5) | - |
| Afternoon | 38 (51.4) | 14 (56.0) | 13 (65.0) | 11 (37.9) | 0.18 |
| Evening | 23 (31.1) | 7 (28.0) | 6 (30.0) | 10 (34.5) | 0.95 |
| Multiple^e^ | 29 (39.2) | 8 (32.0) | 9 (45.0) | 12 (41.4) | 0.69 |
| All Day | 8 (10.8) | 4 (16.0) | 3 (15.0) | 1 (3.5) | 0.23 |
| Total Days of Operation: |  |  |  |  |  |
| 1 Day | 27 (36.5) | 8 (32.0) | 6 (30.0) | 13 (44.8) | 0.55 |
| 2-7 Days | 23 (31.1) | 10 (40.0) | 5 (25.0) | 8 (27.6) |  |
| 8+ Days | 24 (32.4) | 7 (28.0) | 9 (45.0) | 8 (27.6) |  |
| First Date of Operation: |  |  |  |  | 0.02 |
| Day 1 - 3 | 28 (37.8) | 9 (36.0) | 10 (50.0) | 9 (31.0) |  |
| Day 4 - 6 | 26 (35.1) | 12 (48.0) | 8 (40.0) | 6 (20.7) |  |
| Day 7 - 15 | 20 (27.0) | 4 (16.0) | 2 (10.0) | 14 (48.3) |  |
| Site Type: |  |  |  |  |  |
| Community Center | 22 (29.7) | 9 (36.0) | 6 (30.0) | 7 (24.1) | 0.38 |
| Church | 20 (27.0) | 4 (16.0) | 5 (25.0) | 11 (37.9) |  |
| School/Park/Public Street | 18 (24.3) | 7 (28.0) | 3 (15.0) | 8 (27.6) |  |
| Food Retailer/Other^f^ | 14 (18.9) | 5 (20.0) | 6 (30.0) | 3 (10.3) |  |
|  |  |  |  |  |  |
| *Site Resources^d^* |  |  |  |  |  |
| Prepared Meals | 66 (89.2) | 24 (96.0) | 16 (80.0) | 26 (89.7) | 0.28 |
| MREs | 7 (9.5) | 3 (12.0) | 3 (15.0) | 1 (3.5) | 0.36 |
| Groceries | 9 (12.2) | 2 (8.0) | 2 (10.0) | 5 (17.2) | 0.67 |
| Water | 28 (37.8) | 7 (28.0) | 11 (55.0) | 10 (34.5) | 0.18 |
| Ice | 10 (13.5) | 4 (16.0) | 4 (20.0) | 2 (6.9) | 0.36 |

Abbreviations: NH, Non-Hispanic; MRE, meals ready-to-eat.

1. Data presented in this table represents aggregate information on emergency food distribution sites that operated in the two weeks after Hurricane Ida (August 30 – September 13, 2021).
2. *P* values calculated with Fisher’s Exact test. Values < 0.05 were considered statistically significant.
3. Socio-demographic data represent estimates of the population in the census tracts where the food distribution sites were located. These data were gathered from the U.S. Census Bureau (% NH Black and % poverty) or the U.S. Department of Agriculture’s Food Access Atlas (low-income/low-access census tract).
4. Site operation and resource data were extracted from the NOLA Ready public assistance calendar. Variables representing site operations and resources are not mutually exclusive.
5. Multiple indicates that a site was open during two periods or the day: morning/afternoon, morning/evening, or afternoon/evening.
6. Other distribution site includes event/entertainment centers and low-income housing complexes.
